# Could the 2010 HIV outbreak in Athens, Greece have been prevented? A mathematical modeling study

**DOI:** 10.1371/journal.pone.0258267

**Published:** 2021-10-07

**Authors:** Ilias Gountas, Georgios Nikolopoulos, Giota Touloumi, Anastasios Fotiou, Kyriakos Souliotis

**Affiliations:** 1 Faculty of Social and Political Sciences, University of Peloponnese, Korinthos, Greece; 2 Medical School, University of Cyprus, Nicosia, Cyprus; 3 Department of Hygiene, Epidemiology and Medical Statistics, Medical School, National and Kapodistrian University of Athens, Athens, Greece; 4 Greek Reitox Focal Point at the Athens University Mental Health, Neurosciences, & Precision Medicine Research Institute (MHRI), Athens, Greece; Medical University of Warsaw, POLAND

## Abstract

**Introduction:**

In 2009 and 2010, Athens, Greece experienced a hepatitis C virus (HCV) and a Human Immunodeficiency Virus (HIV) outbreak among People Who Inject Drugs (PWID), respectively. The HCV outbreak was not detected, while that of HIV was identified in 2011. The integrated HIV-interventions, launched in early 2012, managed to reduce directly the HIV incidence and indirectly the HCV incidence. This study aims to assess what would have been the course of the HIV outbreak and its associated economic consequences if the 2009 HCV outbreak had been detected and integrated interventions had been initiated 1- or 2-years earlier.

**Methods:**

The model was calibrated to reproduce the observed HIV epidemiological and clinical parameters among PWID of Athens, Greece. We examined the effect of the 1- or 2-years earlier detection scenarios, the 1-year later detection, the non-detection scenario, and compared them to the status quo scenario.

**Results:**

Cumulative HIV cases, under the status-quo scenario during 2009‐2019, were 1360 (90% Credible intervals: 290, 2470). If the HCV outbreak had been detected 1- or 2- years earlier, with immediate initiation of integrated interventions, 740 and 1110 HIV cases could be averted by 2019, respectively. Regarding the costs, if there was an efficient notification system to detect the HCV outbreak 1 or 2 years earlier, 35.2–53.2 million euros could be saved compared to the status quo by 2019.

**Conclusions:**

If the HCV outbreak had been detected and promptly addressed, the HIV outbreak would have been prevented and 35.2–53.2 million euros could have been saved.

## Introduction

People who inject drugs (PWID) are at higher risk of infection with human immunodeficiency virus (HIV) and hepatitis C virus (HCV) compared with the general population [1]. It is estimated that about 8 and 2 million PWID have been infected by HCV and HIV worldwide, respectively [1]. Several studies on PWID have highlighted a strong relationship between HIV and HCV prevalence [2–4]. Specifically, in high HCV prevalence settings (>30%), if HCV prevalence rises, often an increase in HIV prevalence follows shortly [2–5]. Unfortunately, this relationship has been confirmed in Athens, Greece [5]. In 2009 and 2010, Athens, Greece experienced an HCV and a HIV outbreak among PWID, respectively [5].

In 2011, the number of reported cases of HIV among PWID sharply increased (+1250% in 2011 vs 2010), without any changes in testing policy that could explain this alteration [6]. Similarly, the HIV prevalence among PWID of Athens increased from 0.8% to 7.8% between 2010 and 2011 (S1 Fig [7]). The increase in risky behaviors, in combination with the low coverage of existing harm reduction service provision and the severe economic recession, were likely the main drivers behind the emergence of two outbreaks among PWID of Athens, Greece. It is notable that from the two outbreaks (i.e., in 2009 the HCV outbreak and in 2010 the HIV outbreak), only the HIV was detected [5].

Molecular studies have underlined that HIV transmission among PWID in Athens is mostly driven by high-risk injecting [6, 8, 9]. Public health interventions, including the expansion of harm reduction measures and a seek-test and-treat HIV intervention (ARISTOTLE program) [10], were implemented, mainly in 2012, to contain the spread of HIV (since the HCV outbreak was not detected). Those public health responses managed to significantly decrease HIV incidence [11]. Given that HCV and HIV share common transmission routes, HIV interventions managed to reduce HIV incidence by 78.0% and indirectly HCV incidence by 64.8% [5, 11].

An important unstudied hypothesis is whether the 2010 HIV outbreak could have been prevented if the 2009 HCV outbreak had been timely detected. This modeling study aims to assess what would have been the course of the HIV outbreak and the associated economic consequences if the 2009 HCV outbreak had been early identified and the same integrated interventions had been initiated in Athens, Greece in early 2011 or early 2010 instead of 2012.

## Methods

### Description of the HIV mathematical model

A discrete-time, stochastic, individual-based model of HIV transmission among PWID was developed and parameterized to data from Athens (Table 1), with the model structure shown in S2 Fig. The model tracked HIV-susceptible individuals through infection, diagnosis, treatment with antiretroviral therapy (ART), and ART discontinuation. More specifically, the model includes five main mutually exclusive compartments of PWID: 1) Susceptible; 2) Undiagnosed HIV-infected; 3) HIV-infected and diagnosed; 4) Under ART; and 5) PWID with viral suppression. The PWID population was additionally stratified according to risk of infection (high/low-risk), year of infection (1^st^ year/subsequent year), and year on ART (first/second/third/ fourth or more year). Each year, PWID exit the model through death (μ1) or cessation of injection (μ2) and enter the susceptible population at rate θ (set equal to μ1 + μ2 to keep the population size at constant levels). We modeled the force of infection for susceptible PWID to depend on HIV prevalence, on whether PWID are at sharing phase (i.e. sharing phase = high-risk phase; only sharers could be infected), on HIV stages (PWID who are acutely infected have higher transmission probability compared to PWID on the latent stage of infection) and whether PWID have suppressed viral load (having thus lower transmission probability).

**Table 1:** Model parameters and references.

Parameters	Value	References
Size of PWID population (N)	9000	[12]
Proportion of high risk†	23%	[12]
Overall PWID mortality	2%	[13–15]
Duration of injecting career among PWID	12 yrs.	[10]
Duration of initial period of high viremia	3 months	[16]
Cofactor increase in HIV transmission probability during Initial acute phase of high viremia	14.5	[17–19]
ART discontinuation rate per year	6%	[20, 21]
Relative injection-related transmissibility while on ART compared to latent phase*	50%	[16, 22]
Probability of Virologic suppression under ART per year	First year: 74%	Athens Multicenter AIDS Cohort Study data. Kaplan–Meier curve could be found in the appendix (S3 Table, S16 Fig)
	Second year: 85%	
	Third year: 94.5%	
	Fourth year and above: 100% (assumption)	

†Defined as the proportion of PWID who share their injection equipment in the last 30 days.

* Latent phase: The period after the first 3 months since infection during which the infected persons have high levels of viremia.

After infection, PWID progress to the undiagnosed stage. Due to higher viremia levels, PWID in the acute phase have higher probability of transmission through injection than those who are in the latent phase (i.e., The period after the first 3 months since infection) [17–19]. To receive antiviral treatment, PWID should first be diagnosed. Every year, a time-dependent number of PWID are diagnosed and progress to the “infected and diagnosed” compartment of the model. When a PWID is diagnosed, he/she can progress to the ART compartment. ART can lead to virological suppression with a probability pVR. PWID with viral suppression have a lower transmission probability than PWID without virological response. We assumed that ART reduced the risk of transmission via needle/syringe sharing by 50% [16, 22]. The absolute number of PWID on ART before the outbreak was low due to the limited HIV circulation among this population in that period [6, 7] (S1 Table).

To include heterogeneity, the population was additionally stratified by sharing status (sharer or non-sharer. Sharers were defined as the PWID who shared their injection equipment in the last 30 days [12]). Initially, all new injectors are classified as sharers, since it has been shown that PWID are at higher risk of HIV infection during the first years of their injecting career [23, 24]. PWID can cycle from low to high risk (i.e., no sharers, sharers).

### Model calibration

The model was calibrated to match the trajectories of a) HIV prevalence, b) new diagnoses, and c) new ART initiations among PWID in Athens over the period 2002–2019 [7] (S1 Table, S3 Fig). The infection rate, the probability of being diagnosed, and the probability of ART initiation varied until the model reproduced the observed HIV epidemic of Athens (S4–S6 Figs).

For each scenario, 1000 runs were performed (S6 Fig). In order to represent uncertainty in model projections (stochastic variability), the median and 5th and 95th percentiles are shown. Details about the description of the model, the calibration procedure, and the calibration data are available in the Appendix in S1 File.

### Examined scenarios

A ‘status quo’ scenario was used to generate predictions regarding the observed course of the outbreak. Two significant changes happened in the behavior of PWID of Athens, Greece during 2002‐2019. The first was in 2009 when the high-risk behaviors significantly increased [5] and the second was in 2012 when the high-risk behaviors were reduced due to the HIV interventions [11].

Four additional scenarios were examined; two scenarios where all interventions were implemented 1- or 2- years before their actual initiation (i.e., infection rate decreased, diagnoses and ART initiations increased in 2010 or 2011 instead of 2012), one scenario where all interventions started 1- year after their observed initiation (i.e., infection rate decreased, diagnoses and ART initiations increased in 2013 instead of 2012), and, finally, a counterfactual scenario in which all HIV-related interventions were removed to estimate how the HIV outbreak would evolve without any interventions (i.e., no reduction in infection rate and no increase in diagnoses and ART initiations). To capture the second-order transmission effects of the HIV outbreak the time horizon of our analysis was 2002–2019.

Direct costs were calculated by multiplying the annual cost for one treated HIV patient by the number of treated patients and the median treatment time. The average annual direct cost per treated patient in Greece in 2017 was €6860 [25] (This estimation contains all the health care resources consumed by HIV-infected patients in Greece).

### Model validation

The validity of the model was assessed by comparing the model’s incidence rate estimate under the status quo scenario, with an external estimation from a sero-conversion study among PWID of Athens [11].

### Sensitivity analysis

A series of univariate sensitivity analyses was undertaken to examine the impact of different model assumptions on cumulative infections caused by the outbreak. These included the impact of shorter/longer average duration of injecting carrier (10, 14 vs. 12 years), higher/lower annual mortality rate (1%, 4% vs. 2%), higher/lower relative injection-related transmissibility while on ART with undetected viral load compared to latent phase with detectable viral load (25%, 75% vs. 50%), larger/smaller PWID population (8000, 10,000 vs. 9000), and greater/smaller proportion of PWID who share injection equipment (15%, 30% vs. 23%) (S2 Table).

## Results

### Model fit assessment

The status quo model accurately captures the overall trajectories in HIV prevalence, new diagnoses, and antiviral treatments during the time horizon of the study (S7A–S7C Fig). Although not calibrated to HIV incidence, S7D Fig shows that the model reproduces observed trends in HCV incidence as well; the HIV incidence rate estimated by the model in 2012 was *7*.*10% (90% CrI*: *1*.*47%*, *13*.*1%)*, while the corresponding estimate based on sero-conversion data was 7.76% (95% Confidence Intervals: 4.60%, 13.11%). Similarly, the HIV incidence rate estimated by the model in 2013 was *0*.*9% (90% CrI*: *0*.*2%*, *1*.*55%)*, while the one based on sero-conversion data was 1.71% (95% Confidence Intervals: 0.55%, 5.31%) [11].

### HIV prevalence trends

**Fig 1:**
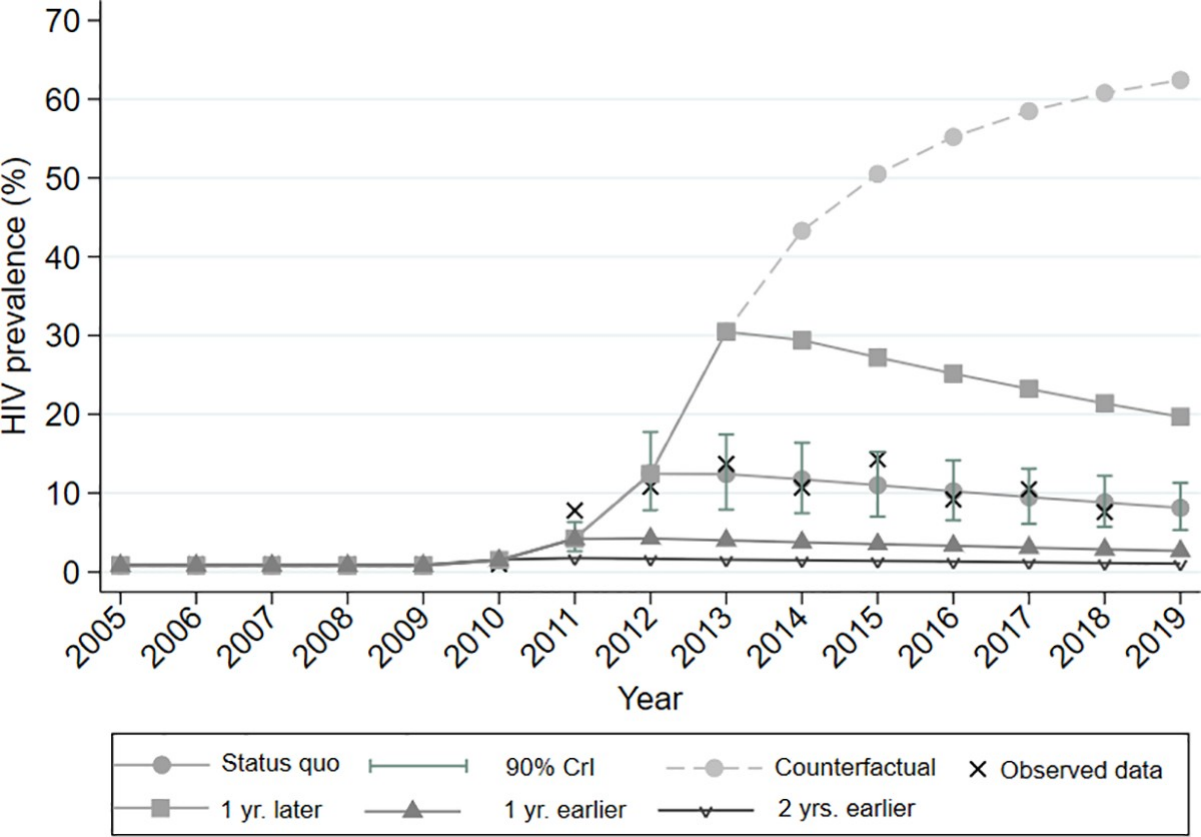
Model predictions for Human Immunodeficiency Virus (HIV) prevalence by the 5 different scenarios (status quo, counterfactual, one-year earlier/later implementation of interventions, and 2-year earlier implementation of interventions). The solid black line and shaded grey error bars show the median and 90% credible intervals (90% CrI) for the model projections. For comparison, asterisks indicate the observed HIV prevalence data.

If the HIV outbreak had been detected in 2011 or 2010, the same public health response would have resulted in relatively reduced, by 55% and 80% respectively, HIV prevalence in 2019, compared to the status quo scenario (Fig 1, S8–S11 Figs). On the contrary, if the interventions had taken place a year later (i.e., 2013), the expected prevalence in 2019 would have been 2.4 times higher than that in the status quo scenario (*19*.*6%* % vs. 8%); Fig 1).

Finally, under the counterfactual scenario, i.e., without any intervention, HIV prevalence would be increasing until 2019 (Fig 1); the estimated HIV prevalence in 2019, under the counterfactual scenario, would be 7.5 times higher compared to the estimated HIV prevalence in 2019 under the status quo scenario (60.5% vs. 8%).

### HIV incidence trends

**Fig 2:**
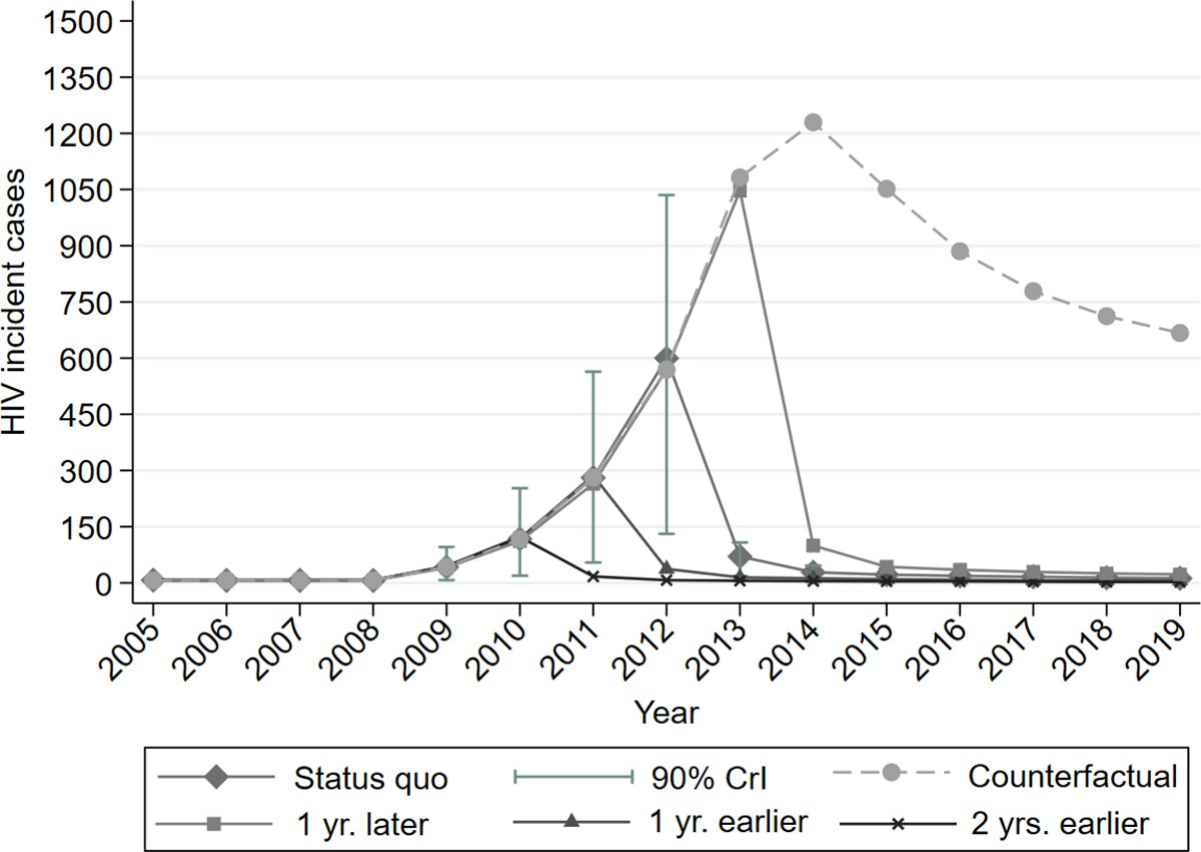
Model predictions for Human Immunodeficiency Virus (HIV) incident cases by the 5 different scenarios (status quo, counterfactual, one-year earlier/later implementation of interventions, and 2-year earlier implementation of interventions scenario). The shaded grey error bars show the 90% credible intervals (90% CrI) for the model projections.

Fig 2 shows the trends in HIV incidence among PWID in Athens. Prior to 2009, the number of new HIV infections were 8 annually (90% Credible intervals (CrI): 1, 17). At the peak of the outbreak (i.e., in 2012), the HIV incidence sharply increased to 600 new cases (90% CrI: 130, 1050). However, after the interventions to control transmission, the annual HIV incidence in 2013 decreased to 70 new infections (90% CrI: 18, 106). It is notable that, despite the successful interventions, the HIV incidence in the post-outbreak was higher than in the pre‐outbreak period (Fig 2). For example, the model estimated that 14 (90% CrI: 3, 20) new infections occurred among the PWID of Athens in 2019.

### Saturation effect of the outbreak

Under the counterfactual scenario, HIV incidence would have increased between 2009–2014 whereas, after 2014, it would have decreased, even in the absence of any intervention, due to the saturation effect of the outbreak. That implies that the large decline in incidence observed after 2011 could be attributed to the HIV interventions, as the counterfactual scenario shows that the saturation effect of the outbreak started after 2014 (Fig 2).

### Additional/Averted cases

The cumulative HIV incident cases in Athens, under the status quo scenario at the end of 2019, were about 1360 (90% CrI: 290, 2470; Fig 2). Under the counterfactual scenario, 7030 (90% CrI: 5240, 8330) additional HIV cases would have occurred during 2009–2019, compared to the status quo scenario (Fig 2, Table 2). Should the HIV outbreak had been detected in 2011 or 2010 with immediate initiation of integrated interventions similar to those implemented in 2012, 740 (90% CrI: -425, 1930) and 1110 (90% CrI: 30, 2250) fewer HIV cases would have been observed compared to the status quo scenario, respectively. Finally, if we had delayed public health responses by 1-year, 1190 (90% CrI: -1000, 3000) additional HIV cases would have occurred during 2009–2019, compared to the status quo scenario (Fig 2, Table 2, S12–S15 Figs).

**Table 2:** Cumulative number of infections and cumulative costs among people who inject drugs during four different scenarios, compared to the status quo scenario. Ranges in the brackets correspond to the 90% credibility intervals.

Scenarios			
	Cumulative number of new infections (90% Credible interval)	Difference compared to the Status quo (90% Credible interval)	Difference in the cumulative costs (millions of euros), compared to the Status quo (90% Credible interval) †
Status quo	1360 (290, 2470)	NA	NA
One-year earlier implementation	620 (120, 1250)	-740 (-1930, +425)	-35.5 (-92.6, +20.4)
Two years earlier implementation	250 (45, 500)	-1110 (-2250, -30)	-53.3 (-108.0, -1.4)
One-year post implementation	2550 (630, 4000)	1190 (-1000, 3000)	57.1 (-48.0, 144.0)
Counterfactual	8390 (6920, 9000)	7030 (5240, 8330)	337.4 (251.5, 399.8)

†Costs is calculated by multiplying the cost of one treatment (6,860 € per treated patient) by the number of the treated patients. The median treatment duration in the model was ~7 years. The time horizon for the table’s estimates is 2009–2019.

### Economic impact

Under the status quo scenario, the direct healthcare costs of the outbreak by 2019 were estimated at 65.3 million euros (90% CrI: 13.9, 118.6). If the HIV outbreak was not detected, the additional cost for the healthcare system would have been 5.16 times higher compared to the status quo [337.4 (90% CrI: 251.5, 565.9) million euros]. On the contrary, if there was an efficient, early warning, notification system so that the public health responses would have been implemented in 2011 or 2010, 53.9% and 81.4% of the estimated cost of the status quo could have been saved (Table 2).

### Sensitivity analysis

**Fig 3:**
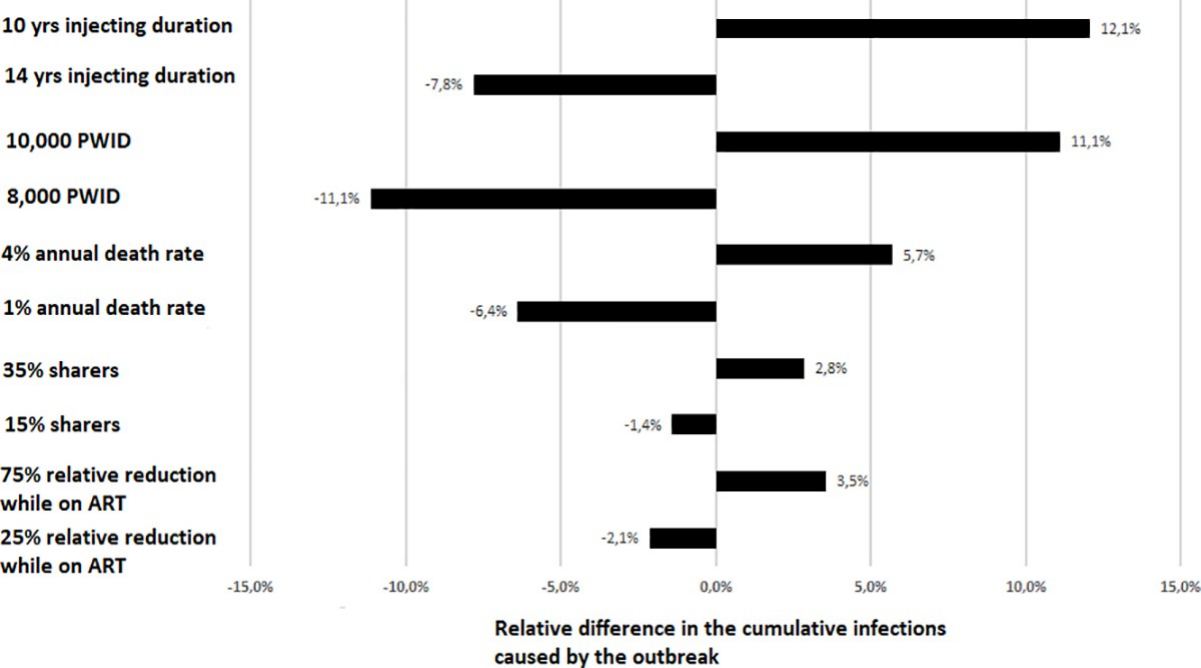
Results of one-way sensitivity analysis showing the relative difference in the cumulative number of new infections caused by the outbreak compared to the base parameter values in Table 1. A value of zero describes no change from the projected cumulative incidence. A positive or a negative value means that the projected incidence is higher or lower, respectively, compared to that projected under the base scenario.

The sensitivity analysis showed that variations in average injecting duration, estimated number of PWID, and annual death rate affected substantially the projected cumulative infections produced by the outbreak. Specifically, under the status quo scenario for a longer injecting duration (14 years instead of 12 years), the cumulative incidence decreased by 7.8%, while for a shorter injection duration (10 years instead of 12 years) it increased by 12.1% (Fig 3).

Compared to the status quo scenario, if the annual death rate was 1% rather than 2%, the cumulative incidence would decrease by 6.4%. On the contrary, if the annual death rate was 4%, the cumulative incidence would increase by 5.7%. Changes in the number of PWID also affected model predictions; if the estimated number of PWID in Athens was 8000 rather than 9000, the cumulative incidence would decrease by 11.1%. If PWID were 10,000, the cumulative incidence would increase by 11.1%. The other assumptions had a marginal impact on the model’s outputs (Fig 3).

## Discussion

Timely detection of an HIV or HCV outbreak is important to prevent further transmission of infections and minimize the magnitude of an outbreak. Our results highlighted that if the HCV outbreak has been detected and promptly addressed, the HIV outbreak would have been almost entirely prevented. This finding supports an important concept regarding public health with significant implications in healthcare policies. HCV transmissibility is higher than that of the HIV, and could be used as a marker of a potential spread of HIV among PWID [2–5]. Our findings add to a growing body of evidence, which supports stronger surveillance systems for PWID to enhance our capacity for early detection of potential outbreaks and timely action [26, 27]. Although the analysis is Athens specific, it could a guide for the management of similar situations in other settings as well. For example, the US Centers for Disease Control and Prevention declared that 220 US counties are at high risk for future HIV or HCV outbreaks among PWID [28].

In most developed countries, monitoring of HIV infection is at a higher priority compared to HCV infection, despite their similar heavy impact on public health. In Greece, the Organization Against Drugs (OKANA) is the sole provider of oral substitution therapy. Developing an automated system of early detection and notification of HIV or HCV outbreaks within the OKANA could be a valuable tool for early action. We found that 740–1110 HIV cases would have been averted and 35.2–53.2 million euros could have been saved by 2019, if there had been an efficient notification system to detect timely the HCV outbreak and responses had been taken timely. Similarly to this study, in our previous analysis, in which we examined the averted cases and the related economic consequences from the HCV perspective, we found that 440–970 HCV cases and 6.8–15.6 million euros could have been saved by 2019 [29]. If we sum the above, our two models showed that a significant number of HIV and HCV cases could be averted and 40.9–65.8 million euros could be saved if there was an efficient notification system to timely detect the HCV/HIV outbreaks.

The results of a delayed response (1-year delay) or no response (counterfactual scenario) were also examined in the analysis. Those scenarios were assessed to underline the high importance of the timely detection of an outbreak. A potential delayed response to an HIV outbreak, even for some months, is expected to increase disease burden and pose significant challenges to the healthcare system (since HIV treatment is lifelong). In the case of the outbreak of Athens, if we had delayed public health responses by 1-year, 1190 additional HIV cases would have occurred compared to the status quo scenario during 2009–2019.

The analysis showed that the significant reductions in HIV incidence in 2012 were fully attributed to the implemented integrated HIV-associated interventions. HIV incidence was still increasing when the public health measures were initiated. The decrease in HIV incidence, as a result of the natural course of the epidemic, would begin, if there had been no response intervention, after 2014. On the contrary, in the case of the HCV outbreak of Athens, from the 64.8% reduction in HCV incidence, it has been demonstrated that 41.5% of this reduction was due to the interventions taken to control the HIV epidemic, while the 23.3% was due to the natural course of the epidemic [5]. The HCV outbreak had a greater saturation effect compared to the HIV outbreak due to differences in the baseline prevalence of the two infections among PWID. In Athens, in 2012, the prevalence of HIV and HCV were 10.8% and 77.1%, respectively. Mathematical models could be a useful tool to disentangle the likely impact of an intervention.

The importance of bridging populations in the dynamic of HIV transmission has been highlighted in many studies [30–32]. PWID could act as an important bridge population as they may transmit HIV infection through sexual transmission and needle-sharing to other high-risk populations or the general population. One of the successes of the HIV interventions implemented in Athens, Greece is that they limited the outbreak mostly within the PWID networks [8]. An efficient notification system among PWID would cause broader benefits to the general population, since not only could protect the PWID population, but at the same time the entire HIV susceptible population.

Wars, economic crises, and disasters that cause significant instability in society, are often referred as “Big events” [33, 34]. Evidence from various settings suggests that Big events are associated with changes in drug use patterns and outbreaks of infectious diseases. This theory was confirmed in Athens, Greece: at the initiation of the severe economic recession in Greece, which was the most recent Big event in the country, an HCV outbreak emerged, which was the root of the HIV outbreak [5, 35, 36]. Nowadays, the COVID-19 pandemic, i.e., the current Big Event, has switched health care priorities and monopolizes the attention of politicians and policymakers [37, 38]. After a Big event, HIV testing and harm reduction interventions should be more intense to prevent new outbreaks among PWID, since it is quite common that surges of HIV or HCV infections in hidden and marginalized populations are detected years after their onset.

### Sensitivity analysis

Models’ estimates are sensitive to their parameters. Sensitivity analyses showed that under a rapid turnover in the population of PWID (i.e., shorter injecting duration or higher death rate), the cumulative infections would be higher compared to the status quo scenario as more PWID would be at risk of infection. The magnitude of the HIV outbreak is strongly dependent on the speed of population renewal. In settings where the population is renewed fast, the cumulative number of infections are higher compared to an outbreak setting where the population is renewed relatively slow. This finding is critical as during crises it is not unusual for the PWID population to be increased for a short time and thus more individuals to be at risk of HCV/HIV infection.

### Limitations

As with any modelling study, there are also limitations. First, the model ignores the impact of social networks on HIV transmission and assumes that the population is totally mixed i.e., sharing injectors have equal contact with all other sharing injectors in the population. Second, the model does not distinguish infections due to injecting drugs or sexual transmission. However, previous analyses have underlined that HIV transmission among PWID in Athens is primarily driven by high-risk injecting [6, 8, 9]. Third, we assumed that all reductions in high-risk behaviors were attributed to the prevention measures (i.e., all the changes in the risky behaviors of the PWID are attributed to the intervention). Fourth, the averted costs of an earlier response have been computed using a simple multiplication method. Future work that will compute the averted costs using more sophisticated methods that would include individual patient’s trajectories along with an estimation of their infection dates is needed. Finally, the model did not take into account the potential for increased coinfection mortality. However, the additional coinfection mortality risk among HIV‐infected PWID is likely to be marginal since the majority of the HIV‐infected PWID were infected recently.

## Conclusions

This paper provides theoretical support that the HIV outbreak could be prevented if the HCV outbreak was timely detected. This finding, although specific to the Athenian setting in Greece, could be used as a guide for the management of similar situations in other settings as well.

## Supporting information

S1 File Appendix. (DOCX)

S1 Fig Observed HIV and HCV prevalence among PWID of Athens. (PDF)

S2 Fig Schematic outline of the mathematical model. (PDF)

S3 Fig Model predictions for HIV prevalence among people who inject drugs (PWID) in Athens, Greece under the status quo scenario. The error bars show the 90% credible intervals (90% CrI) for the status quo scenario. For comparison, asterisks indicate the observed HIV prevalence data.
(PDF)

S4 Fig Model predictions for new HIV diagnoses among people who inject drugs (PWID) in Athens, Greece under the status quo scenario. The error bars show the 90% credible intervals (90% CrI) for the status quo scenario. For comparison, asterisks indicate the observed new HIV diagnoses.
(PDF)

S5 Fig Model predictions for annual treatment initiations among people who inject drugs (PWID) in Athens, Greece under the status quo scenario. The error bars show the 90% credible intervals (90% CrI) for the status quo scenario. For comparison, asterisks indicate the observed cumulative treatment initiations.
(PDF)

S6 Fig Model predictions for HIV prevalence under status quo scenario for the first 20 simulations (different colors) of model. The solid black line shows the median estimation.
(PDF)

S7 Fig Model fit of observed HIV prevalence (A), new HIV diagnoses (B), and cumulative antiviral treatments (C) for calibration, and external validity for HIV incidence (D).
(PDF)

S8 Fig Model predictions for HIV prevalence for status quo scenario and counterfactual scenario. (PDF)

S9 Fig Model predictions for HIV prevalence for status quo scenario and 1-year earlier scenario. (PDF)

S10 Fig Model predictions for HIV prevalence for status quo scenario and 2-year earlier scenario. (PDF)

S11 Fig Model predictions for HIV prevalence for status quo scenario and 1-year later scenario. (PDF)

S12 Fig Estimation of the number of incident cases of HIV infection under the status quo and counterfactual scenario. (PDF)

S13 Fig Estimation of the number of incident cases of HIV infection under the status quo and 1-year earlier scenario. (PDF)

S14 Fig Estimation of the number of incident cases of HIV infection under the status quo and 2-year earlier scenario. (PDF)

S15 Fig Estimation of the number of incident cases of HIV infection under the status quo and 1-year later scenario. (PDF)

S16 Fig Kaplan–Meier curve of the probability of no virologic suppression. (PDF)

S1 Table Calibration parameters. (PDF)

S2 Table Sensitivity analysis table. (PDF)

S3 Table Time to virological response during ART therapy by period. (PDF)
