# Supplementary material for: Could the 2010 HIV outbreak in Athens, Greece have been prevented? A mathematical modeling study
Source: PLoS One. 2021 Oct 7;16(10):e0258267. doi: 10.1371/journal.pone.0258267 (PMC8496824; doi:10.1371/journal.pone.0258267)
Supplement: S1 File — (DOCX) [file pone.0258267.s001.docx]

**Could the 2011 HIV outbreak in Athens, Greece have been prevented? A mathematical modeling study**

Gountas Ilias^1^, Nikolopoulos Georgios^2^, Touloumi Giota^3^, Fotiou Anastasios^4^, Souliotis Kyriakos^1^

1 Faculty of Social and Political Sciences, University of Peloponnese, Korinthos, Greece

2 Medical School, University of Cyprus, Nicosia, Cyprus.

3 Department of Hygiene, Epidemiology and Medical Statistics, Medical School, National and Kapodistrian University of Athens, Athens, Greece

4 Greek Reitox focal point at the Athens University Mental Health, Neurosciences, & Precision Medicine Research Institute (MHRI), Athens, Greece

**Appendix**

Content

[**HIV and Hepatitis C prevalence among PWID of Athens** 2](#_Toc71549701)

[**Schematic outline of the mathematical model for HIV transmission** 3](#_Toc71549702)

[**Model calibration to epidemiological and clinical data** 4](#_Toc71549703)

[Calibration estimates 4](#_Toc71549704)

[HIV prevalence 5](#_Toc71549705)

[New HIV diagnoses & antiretroviral treatment (ART) initiations 7](#_Toc71549706)

[**Model’s type** 9](#_Toc71549707)

[**Goodness-of-fit metric (GoF)** 10](#_Toc71549708)

[**Sensitivity Analysis** 11](#_Toc71549709)

[**Model fit assessment** 12](#_Toc71549710)

[**Detailed projections with credible intervals** 15](#_Toc71549711)

[HIV prevalence 15](#_Toc71549712)

[HIV incident cases 18](#_Toc71549713)

[**Trends in Virological response to ART therapy** 21](#_Toc71549714)

[**References** 22](#_Toc71549715)

# **HIV and Hepatitis C prevalence among PWID of Athens**

**Figure S1.** Observed HIV and HCV prevalence among PWID of Athens [[1](#_ENREF_1)]

# **Schematic outline of the mathematical model for HIV transmission**

**Figure S2.** Schematic outline of the mathematical model for HIV transmission (A) and (B) behavioral states among People Who Inject Drugs (PWID). PWID begin as susceptible to infection. Once infected, they progress to the undiagnosed infected compartment and then to the diagnosed infected compartment. Diagnosed cases may enter the healthcare system and start antiretroviral therapy. Individuals who achieve virological response have a lower probability to transmit HIV. Every year, some PWID could be lost to follow-up and return to the “diagnosed and nonlinked to care” status. Finally, individuals can cycle from low to high risk.


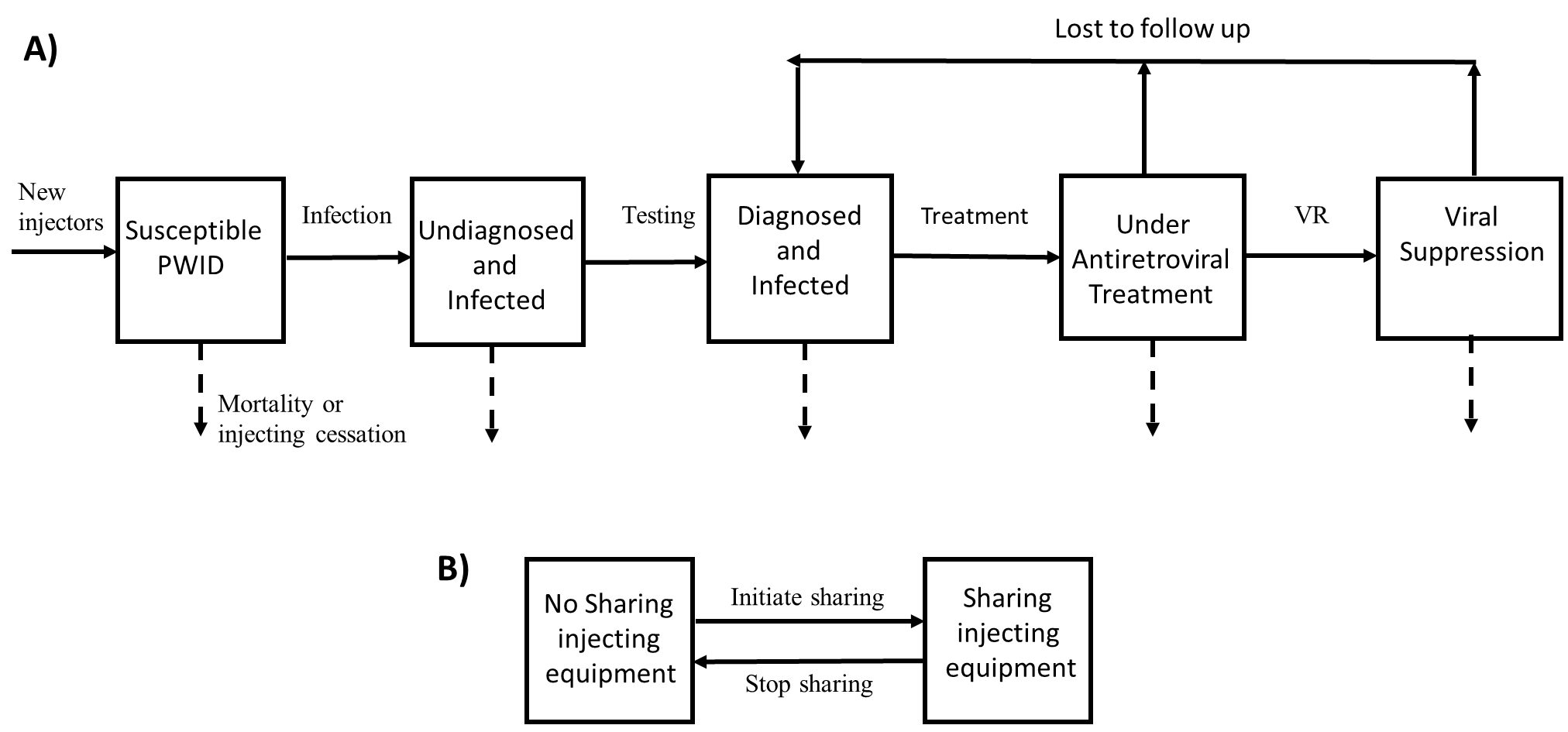


# **Model calibration to epidemiological and clinical data**

Fitting a model to empirical data (calibration procedure) improves the confidence that the simulation model provides a realistic and accurate estimate of the outcome for health policy decisions. Thus, we provided a detailed calibration procedure for our model. More specifically, the model was calibrated against observed HIV prevalence, annual HIV diagnoses, and antiretroviral treatment (ART) initiations among PWID over time. Methodology and comparisons for each calibration point are shown in the following sections.

The simulations were performed in the low-level programming language C++ (Dev-C++ v.5.11) and the graphs were produced in Stata 16.1.

## Calibration estimates

Calibration estimates were retrieved from the 2020 Annual report on the situation of the problem of drugs and alcoholics in Greece from the Greek Reitox focal point.

**Table S1.** Calibration parameters

| Year |  |  |  |
| --- | --- | --- | --- |
|  | **HIV prevalence (%)** | **New diagnoses** | **New ART initiation** |
| 2006 | 0,5 | 17 | 5 |
| 2007 | 0,5 | 11 | 5 |
| 2008 | 0,5 | 11 | 5 |
| 2009 | 0,7 | 15 | 5 |
| 2010 | 0,8 | 16 | 10 |
| 2011 | 7,8 | 266 | 64 |
| 2012 | 10,8 | 551 | 186 |
| 2013 | 13,7 | 272 | 236 |
| 2014 | 10,7 | 106 | 188 |
| 2015 | 14,3 | 70 | 145 |
| 2016 | 9,2 | 81 | 127 |
| 2017 | 10,5 | 86 | 112 |
| 2018 | 7,6 | 106 | 60 |
| 2019 |  | 72 | 53 |

## HIV prevalence

The infection rate (IR) varied until the model reproduced the observed HIV prevalence in Athens, Greece. The IR is a time‐dependent variable. The model highlighted that two significant changes happened in the behavior of PWID living in Athens during 2002-2019. The first was in 2009 indicating the emergence of the outbreak and the second is 2012 reflecting the effect of HIV interventions. Thus, to optimize the fit of the model we used 3 levels of IR (λ1 pre outbreak injecting risk behavior, λ2 during outbreak injecting risk behavior, and λ3 post-outbreak injecting risk behavior). Schematic representation is in Figure S1. We assumed that prior to 2002, the epidemic was at steady state (the level of HIV prevalence in the population of PWID was almost constant; equilibrium state).

$$\lambda=\left\{ \begin{aligned} \lambda1, & year\leq2008 \\ \lambda2, & year\geq2009 AND year\leq2011 \\ \lambda3, year>2011 \end{aligned} \right.$$

To estimate the optimal combination of parameters, a two-step repetitive process was used. Initially, a combination of the parameters that fit well the observed prevalence (λ1, λ2, λ3) was manually found and then through an iterative process the estimates that highly reflected epidemiologic data were identified. Then, we examined all the possible combinations ranged between the ±20% of the initial values. We assessed 10800 different parameter sets. To assess which set of parameters provides the optimal fit to the prevalence data, the weighted least square method was used (minimize the square distance between observed and modeled data).

**Figure S3.** Model predictions for HIV prevalence among people who inject drugs (PWID) in Athens, Greece under the status quo scenario. The error bars show the 90% credible intervals (90% CrI) for the status quo scenario. For comparison, cycles indicate the observed HIV prevalence data.

**Modelling the increased infectivity during the acute phase**

Due to higher HIV viremia levels during acute phase, PWID in the acute phase have higher probability of transmission through injection than those who are longer-term infected [[2-4](#_ENREF_2)]. The model has been constructed using yearly time steps. Thus, we had to assume that the probability of being infected is homogeneously distributed within the year. To model the increased infectivity of a newly infected PWID (during the first 3 months since infection), we assumed that a) during the phase of high viremia, PWID is 14.5 times more infectious than PWID in the latent phase and b) this high viremia lasts for the 3 first months since infection. Based on these assumptions, we implemented the following calculation:

3/12 (3 out of the 12 months) *14.5 (Cofactor increase in HIV transmission during Initial acute phase) + 9/12 (9 out of 12 months) *1 (Cofactor increase in HIV transmission during the latent phase) = 4.375

The above calculation leads to assuming that a newly infected PWID (at his/her 1^st^ year of infection) had a 4.375 times higher probability of transmitting the disease compared to a PWID who had been infected for more than a year.

## New HIV diagnoses & antiretroviral treatment (ART) initiations

Similarly, to the used methodology to calibrate HIV prevalence, a three-level variable was used to calibrate the diagnoses.

**Figure S4.** Model predictions for new HIV diagnoses among people who inject drugs (PWID) in Athens, Greece under the status quo scenario. The error bars show the 90% credible intervals (90% CrI) for the status quo scenario. For comparison, x’s indicate the observed new HIV diagnoses. d1, d2, d3 are the probabilities of being diagnosed

**Figure S5.** Model predictions for annual treatment initiations among people who inject drugs (PWID) in Athens, Greece under the status quo scenario. The error bars show the 90% credible intervals (90% CrI) for the status quo scenario. For comparison, x’s indicate the observed cumulative treatment initiations. T1, T2, T3 are the probabilities of initiating ART.

# **Model’s type**

In our analysis, a discrete-time, stochastic, individual-based model (IBM) was used. IBM simulates the patients’ trajectories at an individual level. It is important that those models possess some inherent randomness due to their methodology. The way that the model examines if a pseudo-individual changes state (e.g., from susceptible to infected) is through the draw of random numbers. More specifically, the model estimates the probability of moving from one stage to the next (e.g., from susceptible to infected). Then for each pseudo-individual, a random number from a Uniform (0,1) distribution is drawn. If the resulted random number (e.g., 0.3) is smaller than the estimated probability of changing stage (e.g., 0.4), then this pseudo-individual changes stage and vice versa. For example, regarding the transmission from susceptible to infected, if the risk of infection is 20%, then all the individuals with drawn random numbers lying in the range of (0-0.2) are assumed to become infected. As the outcome of each run depends on chance, every simulation leads to slightly different results. Uncertainty comes from a single set of parameters but across multiple simulations with randomness included. For that, results over all simulations are pooled and the median along a range is normally presented (stochastic variability). In those models, in order for the results to be reliable, several runs should be conducted since if the number of runs is limited, extreme results from simulations would affect significantly the pooled estimates. For more details regarding the IBM models, please look at [[5](#_ENREF_5)].

**Figure S6.** Model predictions for HIV prevalence under the status quo scenario for the first 20 simulations (different colors) of model. The solid black line shows the median estimation.

# **Goodness-of-fit metric (GoF)**

A GoF metric assists as the objective function in an optimization procedure, measuring the accuracy of the model’s predictions against the targets. In our model, the weighted least square method was used to measure the accuracy. The weighting factors leave preferences on the set of the evaluated targets. The weighting factors are judged on the basis of the relative importance of those targets.

GoF_overall_=∑ (w_prevalence*_ (Modelled prevalence -observed prevalence)^2^ + w_diagnosis*_ (Modelled diagnoses -observed diagnoses)^2^ + w_treatment*_ (Modelled treatments -observed treatments)^2^)

We rank each target equally important with respect to the model's fit. Thus, we gave a value of 0.33 weight in all targets. Smaller values of the GoF metric indicate a better fit to the observed data.

# **Sensitivity Analysis**

To examine the impact of different model assumptions on cumulative infections caused by the outbreak, we undertook a series of univariate sensitivity analyses, displayed in Table S2.

**Table S2.** Sensitivity Analysis table.

| **Parameters** | **Value used in the primary analysis** | **Values examined in the sensitivity analysis** |
| --- | --- | --- |
| **Duration of injecting carrier among PWID in Athens** | 12 years | 10 or 14 years |
| **Overall PWID mortality per annum** | 2% | 1% or 4% |
| **Injection-related transmissibility while on ART compared to latent phase** | 50% | 25% or 75% |
| **Proportion of sharers PWID** | 23% | 15% or 30% |
| **PWID population size in Athens** | 9000 | 8000 vs 10000 |
| **ART discontinuation rate per year** | 6.5% | 3% or 10% |

# **Model fit assessment**

The model has been calibrated to HIV prevalence/new diagnoses/treatment initiations data and validated using the incidence results from the Sypsa et al.

**Figure S7.** Model fit of observed HIV prevalence (A), new HIV diagnoses (B), and cumulative antiviral treatments (C) for calibration, and external validity for HIV incidence (D). For the graph D, the solid black line and shaded grey areas show the median and 90% credible intervals (90% CrI) for the model projections. For comparison, asterisks indicate the observed HIV data while the square indicate the estimated HIV incidence by Sypsa et al. [[6](#_ENREF_6)].

1. HIV prevalence

1. New HIV diagnoses

1. Cumulative treatments

1. HIV incidence rate

# **Detailed projections with credible intervals**

In our main text, we included credible intervals (CrIs) only for the status quo scenario. If we had included all the CrIs for all the examined scenarios the images would be hard to read. Thus, below we present all of our scenarios compared to the status quo with all the CrIs.

## HIV prevalence

**Figure S8.** Model predictions for HIV prevalence for status quo scenario and counterfactual scenario.

**Figure S9.** Model predictions for HIV prevalence for status quo scenario and 1-year earlier scenario.

**Figure S10.** Model predictions for HIV prevalence for status quo scenario and 2-year earlier scenario.

**Figure S11.** Model predictions for HIV prevalence for status quo scenario and 1-year later scenario.

## HIV incident cases

**Figure S12.** Estimation of the number of incident cases of HIV infection under the status quo and counterfactual scenario.

**Figure S13.** Estimation of the number of incident cases of HIV infection under the status quo and 1-year earlier scenario.

**Figure S14.** Estimation of the number of incident cases of HIV infection under the status quo and 2-year earlier scenario.

**Figure S15.** Estimation of the number of incident cases of HIV infection under the status quo and 1-year later scenario

# **Trends in Virological response to ART therapy**

Through the Athens Multicenter AIDS Cohort Study (AMACS) data we estimated the time (in months) from the diagnosis date to first date with Viral Load (VL)<=500copies/ml, censored at the date of last viral load measurement. We ignored PWID with VL<=500copies/ml before ART initiation (i.e., considered as non-suppressed).

**Table S3.** Time to virological response during ART therapy by period.

| Time | Total | Fail | Survivor Function | 95% Confidence Intervals |
| --- | --- | --- | --- | --- |
| 12 | 130 | 402 | 0.2607 | 0.2243, 0.2984 |
| 24 | 67 | 55 | 0.1451 | 0.1169, 0.1784 |
| 36 | 27 | 33 | 0.0646 | 0.0477, 0.0944 |

**Median time:** 50% of the PWID achieve virologic suppression in 4.2 months from the ART initiation

**Figure S16.** Kaplan–Meier curve of the probability of no virologic suppression

# **References**

1. Greek Reitox focal point. Annual report on the situation of the problem of drugs and alcoholics in greece (in Greek). 2020.

2. Boily MC, Baggaley RF, Wang L, Masse B, White RG, Hayes RJ, et al. Heterosexual risk of HIV-1 infection per sexual act: systematic review and meta-analysis of observational studies. The Lancet Infectious diseases. 2009;9(2):118-29.

3. Borquez A, Beletsky L, Nosyk B, Strathdee SA, Madrazo A, Abramovitz D, et al. The effect of public health-oriented drug law reform on HIV incidence in people who inject drugs in Tijuana, Mexico: an epidemic modelling study. The Lancet Public health. 2018;3(9):e429-e37.

4. Morgan D, Mahe C, Mayanja B, Okongo JM, Lubega R, Whitworth JA. HIV-1 infection in rural Africa: is there a difference in median time to AIDS and survival compared with that in industrialized countries? Aids. 2002;16(4):597-603.

5. Vynnycky Emilia , Richard White An Introduction to Infectious Disease Modelling Illustrated Edition, Kindle Edition2010.

6. Sypsa V, Psichogiou M, Paraskevis D, Nikolopoulos G, Tsiara C, Paraskeva D, et al. Rapid Decline in HIV Incidence Among Persons Who Inject Drugs During a Fast-Track Combination Prevention Program After an HIV Outbreak in Athens. The Journal of infectious diseases. 2017;215(10):1496-505.
