# Supplementary figures and images for: Could the 2010 HIV outbreak in Athens, Greece have been prevented? A mathematical modeling study
Source: PLoS One. 2021 Oct 7;16(10):e0258267. doi: 10.1371/journal.pone.0258267 (PMC8496824; doi:10.1371/journal.pone.0258267)

**Figure S1.** Observed HIV and HCV prevalence among PWID of Athens [1]

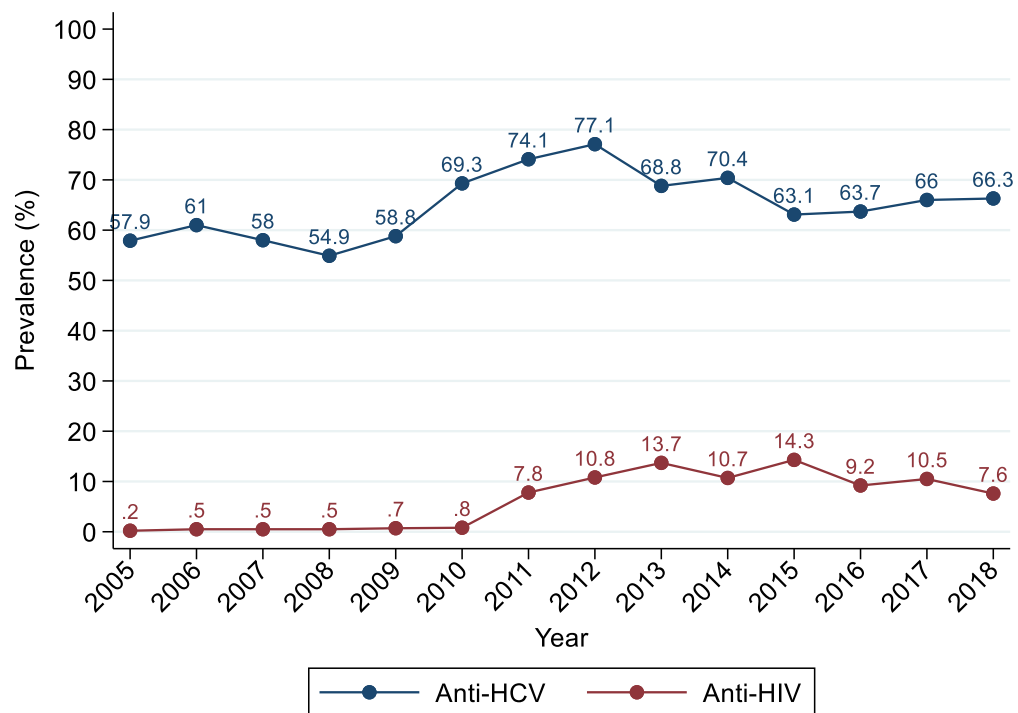

Supplement: S1 Fig — (PDF) [file pone.0258267.s002.pdf]

**Figure S11.** Model predictions for HIV prevalence for status quo scenario and 1-year later scenario.

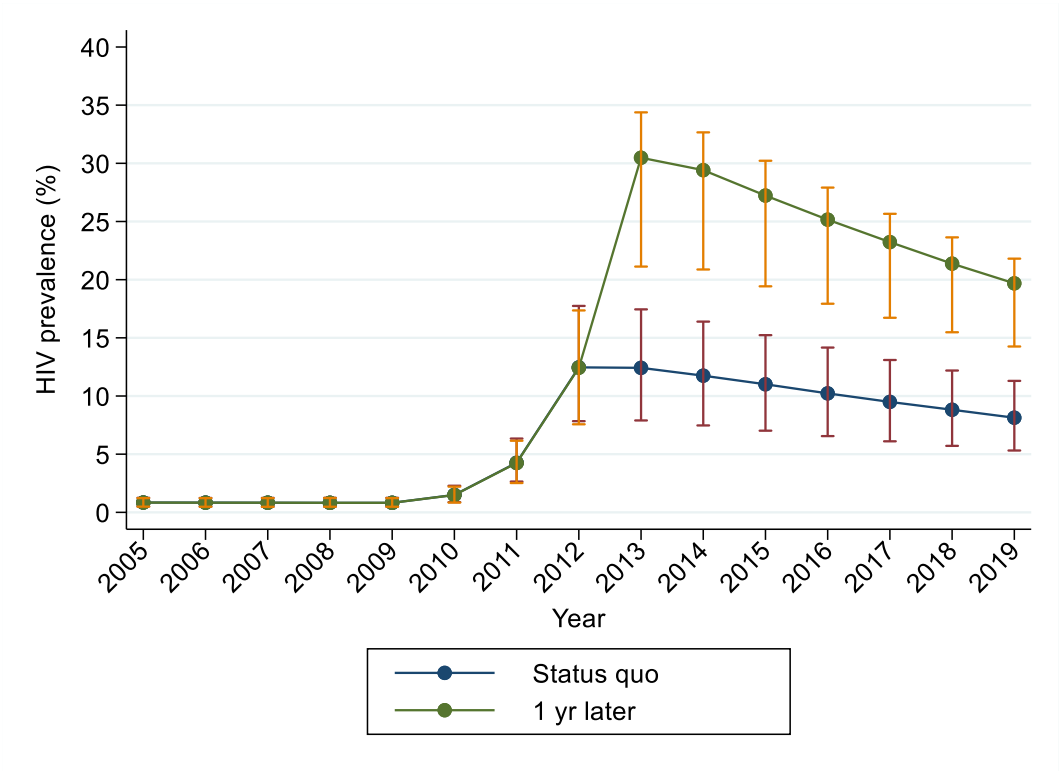

Supplement: S11 Fig — (PDF) [file pone.0258267.s012.pdf]

**Figure S16.** Kaplan–Meier curve of the probability of no virologic suppression

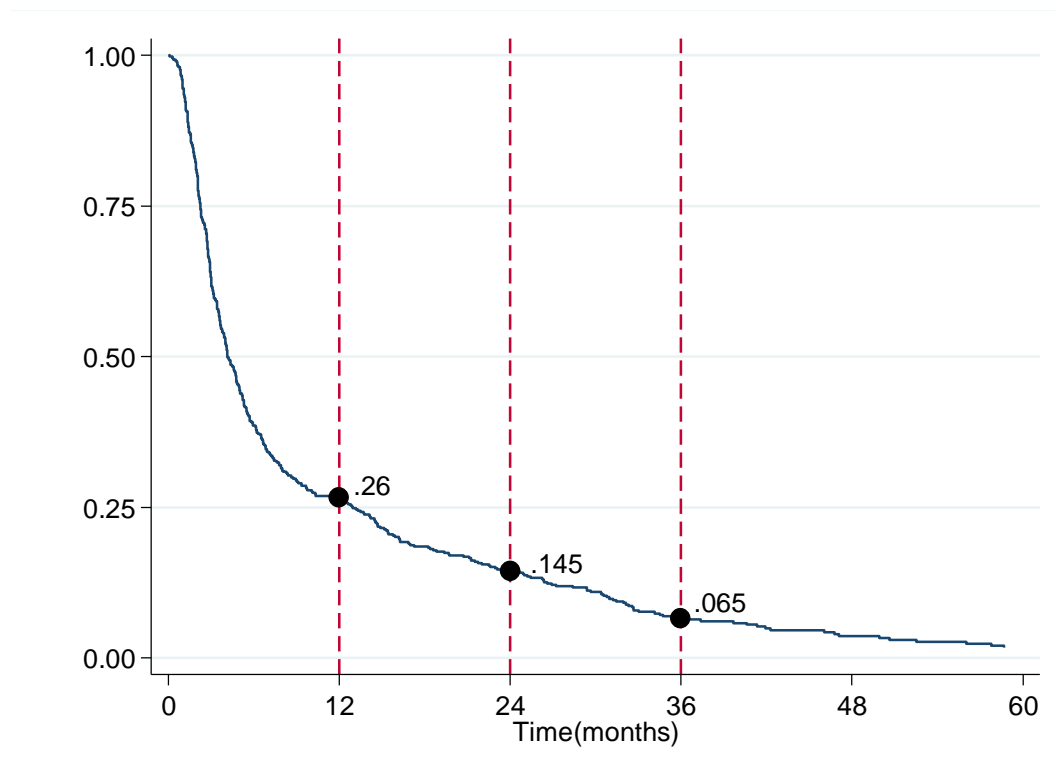

Supplement: S16 Fig — (PDF) [file pone.0258267.s017.pdf]
