# Supplementary material for: Could the 2010 HIV outbreak in Athens, Greece have been prevented? A mathematical modeling study
Source: PLoS One. 2021 Oct 7;16(10):e0258267. doi: 10.1371/journal.pone.0258267 (PMC8496824; doi:10.1371/journal.pone.0258267)

Figure S2. Schematic outline of the mathematical model for HIV transmission (A) and (B) behavioral states among People Who Inject Drugs (PWID). PWID begin as susceptible to infection. Once infected, they progress to the undiagnosed infected compartment and then to the diagnosed infected compartment. Diagnosed cases may enter the healthcare system and start antiretroviral therapy. Individuals who achieve virological response have a lower probability to transmit HIV. Every year, some PWID could be lost to follow-up and return to the "diagnosed and nonlinked to care" status. Finally, individuals can cycle from low to high risk.

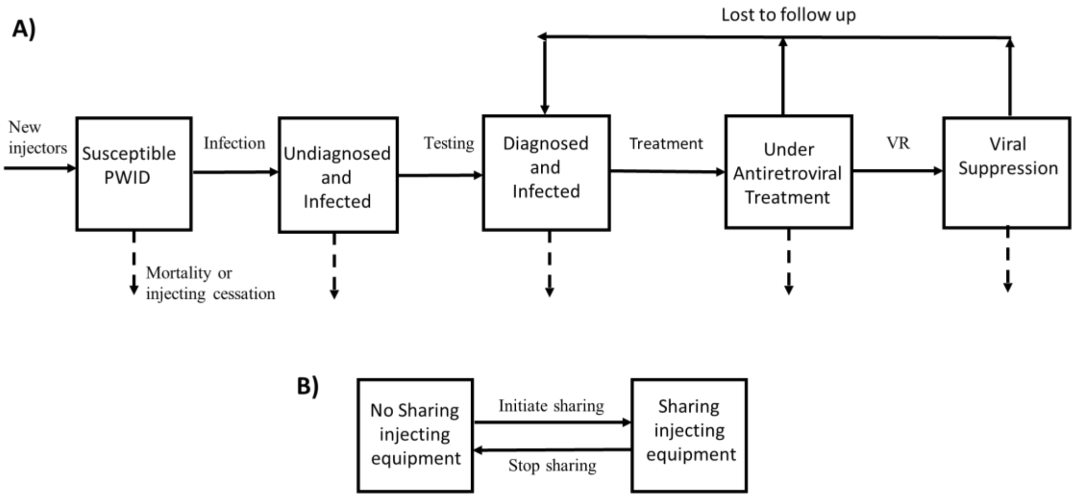

Supplement: S2 Fig — (PDF) [file pone.0258267.s003.pdf]
