# Supplementary material for: Could the 2010 HIV outbreak in Athens, Greece have been prevented? A mathematical modeling study
Source: PLoS One. 2021 Oct 7;16(10):e0258267. doi: 10.1371/journal.pone.0258267 (PMC8496824; doi:10.1371/journal.pone.0258267)

Figure S3. Model predictions for HIV prevalence among people who inject drugs (PWID) in Athens, Greece under the status quo scenario. The error bars show the 90% credible intervals (90% CrI) for the status quo scenario. For comparison, cycles indicate the observed HIV prevalence data.

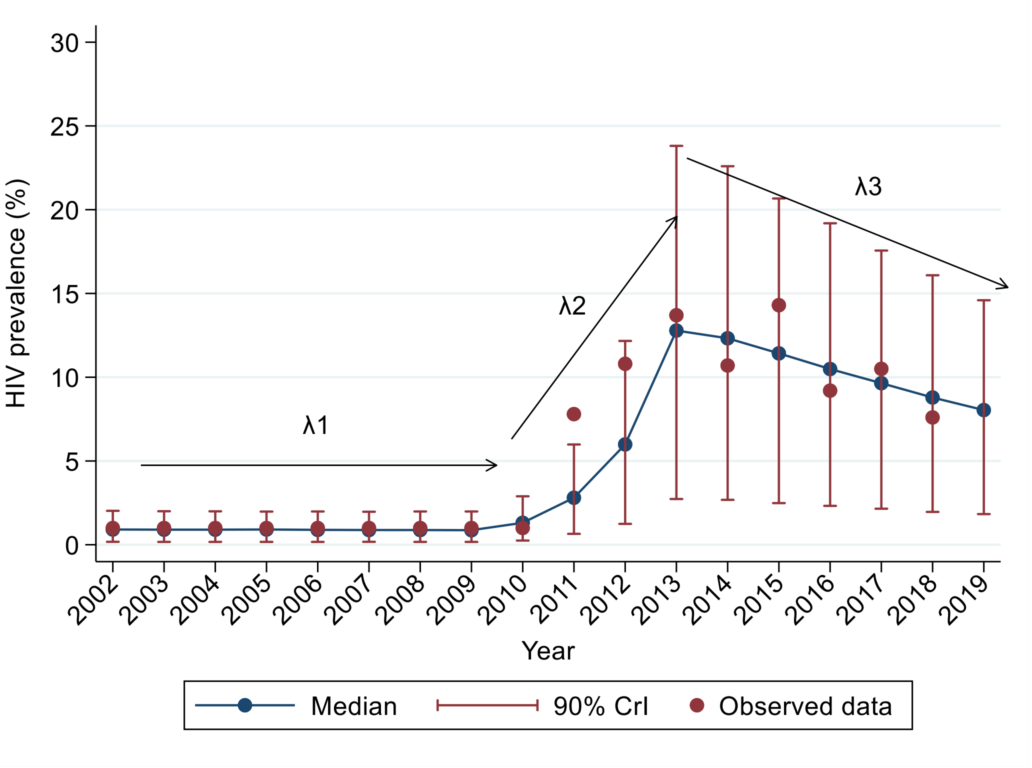

Supplement: S3 Fig — The error bars show the 90% credible intervals (90% CrI) for the status quo scenario. For comparison, asterisks indicate the observed HIV prevalence data. (PDF) [file pone.0258267.s004.pdf]
