# Supplementary material for: Could the 2010 HIV outbreak in Athens, Greece have been prevented? A mathematical modeling study
Source: PLoS One. 2021 Oct 7;16(10):e0258267. doi: 10.1371/journal.pone.0258267 (PMC8496824; doi:10.1371/journal.pone.0258267)

Figure S5. Model predictions for annual treatment initiations among people who inject drugs (PWID) in Athens, Greece under the status quo scenario. The error bars show the 90% credible intervals (90% CrI) for the status quo scenario. For comparison, x's indicate the observed cumulative treatment initiations. T1, T2, T3 are the probabilities of initiating ART.

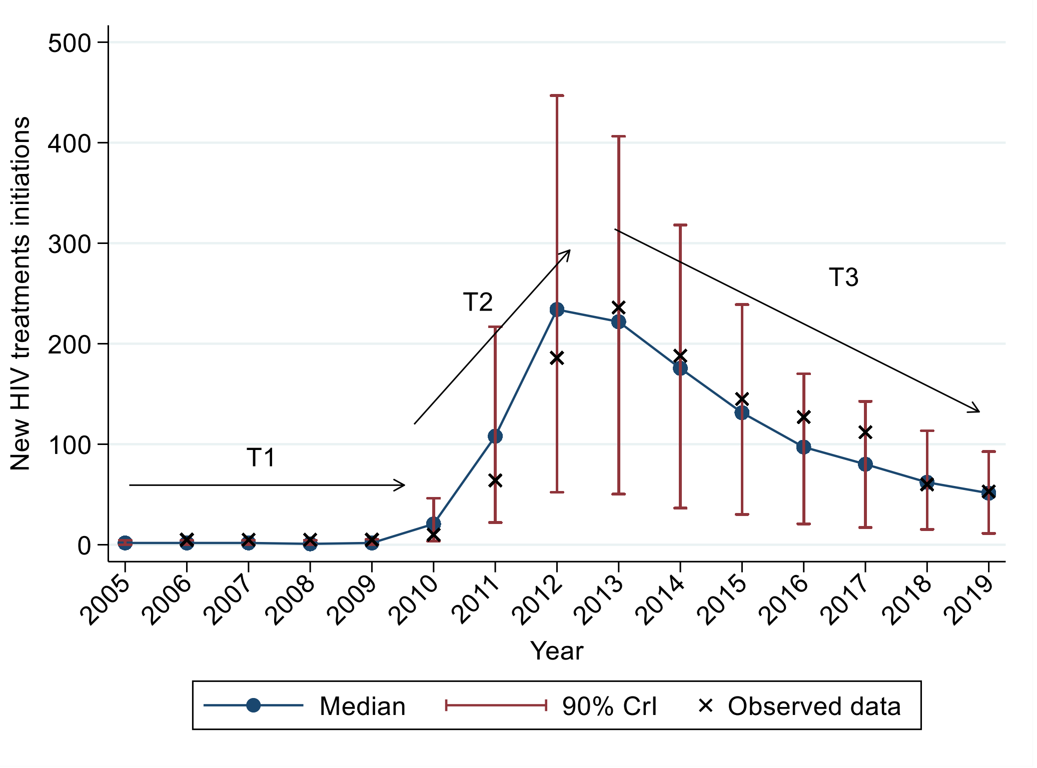

Supplement: S5 Fig — The error bars show the 90% credible intervals (90% CrI) for the status quo scenario. For comparison, asterisks indicate the observed cumulative treatment initiations. (PDF) [file pone.0258267.s006.pdf]
