# Supplementary material for: Could the 2010 HIV outbreak in Athens, Greece have been prevented? A mathematical modeling study
Source: PLoS One. 2021 Oct 7;16(10):e0258267. doi: 10.1371/journal.pone.0258267 (PMC8496824; doi:10.1371/journal.pone.0258267)

Figure S6. Model predictions for HIV prevalence under the status quo scenario for the first 20 simulations (different colors) of model. The solid black line shows the median estimation.

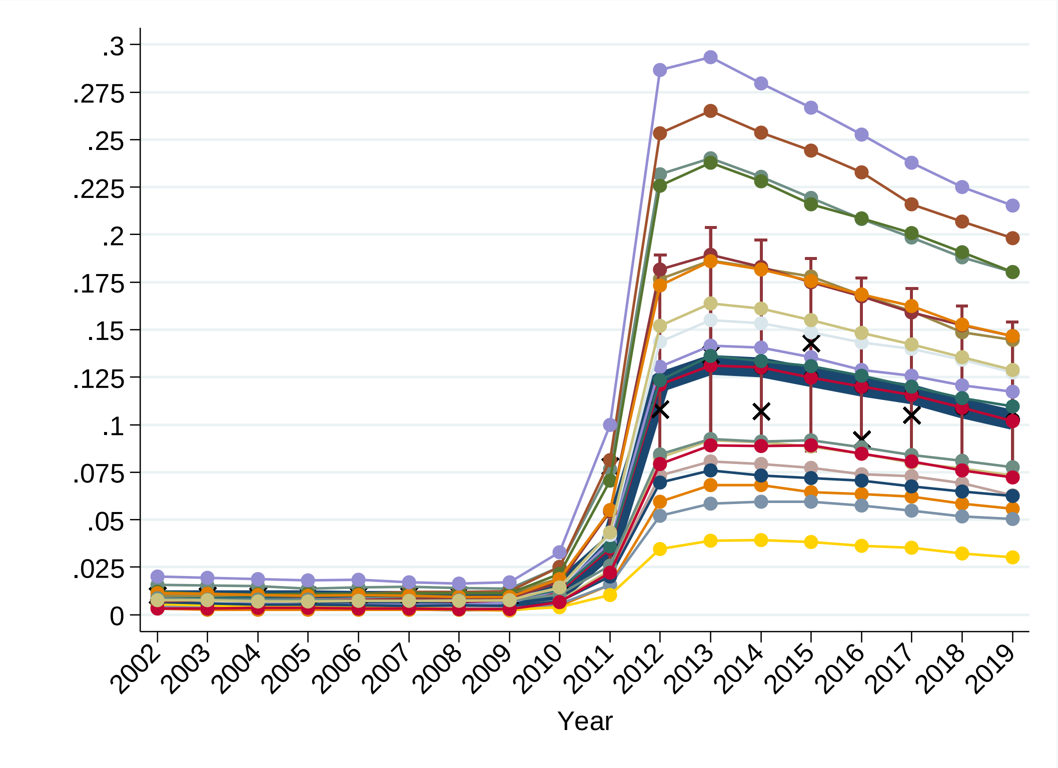

Supplement: S6 Fig — The solid black line shows the median estimation. (PDF) [file pone.0258267.s007.pdf]
