# Supplementary material for: Could the 2010 HIV outbreak in Athens, Greece have been prevented? A mathematical modeling study
Source: PLoS One. 2021 Oct 7;16(10):e0258267. doi: 10.1371/journal.pone.0258267 (PMC8496824; doi:10.1371/journal.pone.0258267)
Supplement: S7 Fig — Model fit of observed HIV prevalence (A), new HIV diagnoses (B), and cumulative antiviral treatments (C) for calibration, and external validity for HIV incidence (D). (PDF) [file pone.0258267.s008.pdf]

Figure S7. Model fit of observed HIV prevalence (A), new HIV diagnoses (B), and cumulative antiviral treatments (C) for calibration, and external validity for HIV incidence (D). For the graph D, the solid black line and shaded grey areas show the median and 90% credible intervals (90% CrI) for the model projections. For comparison, asterisks indicate the observed HIV data while the square indicate the estimated HIV incidence by Sypsa et al. [6].

A) HIV prevalence

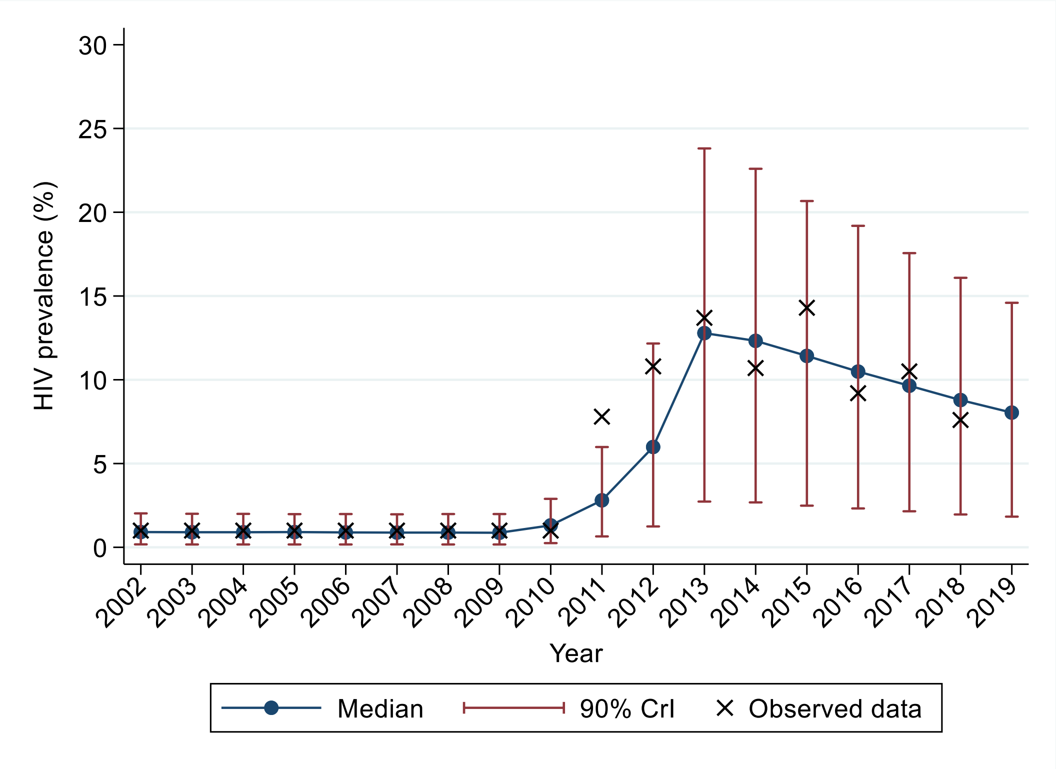

B) New HIV diagnoses

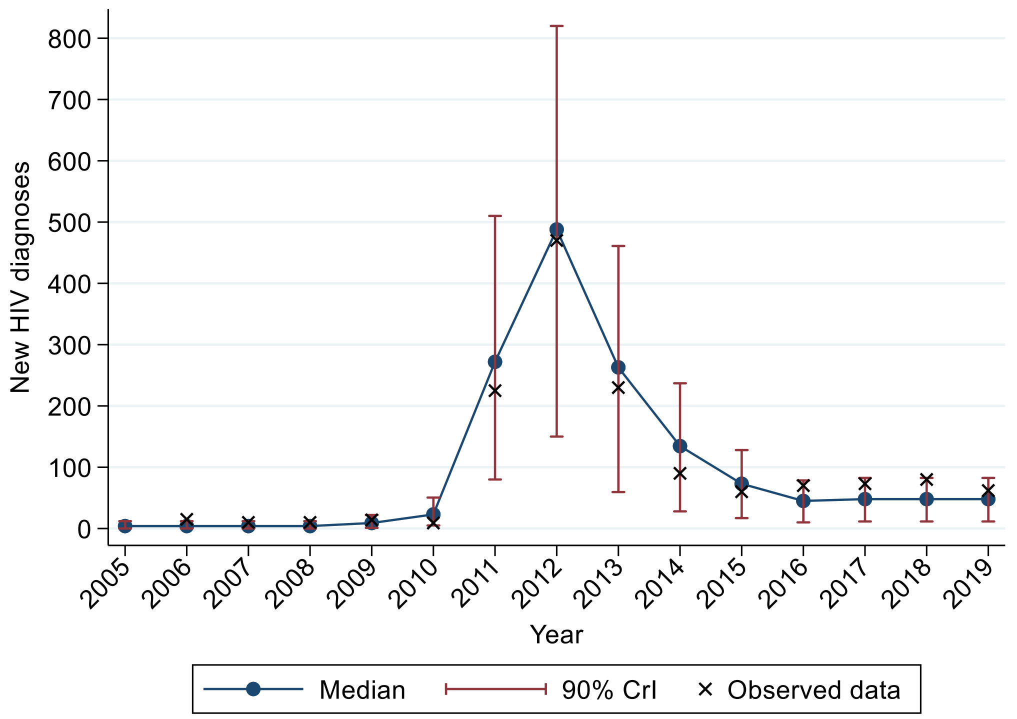

C) Cumulative treatments

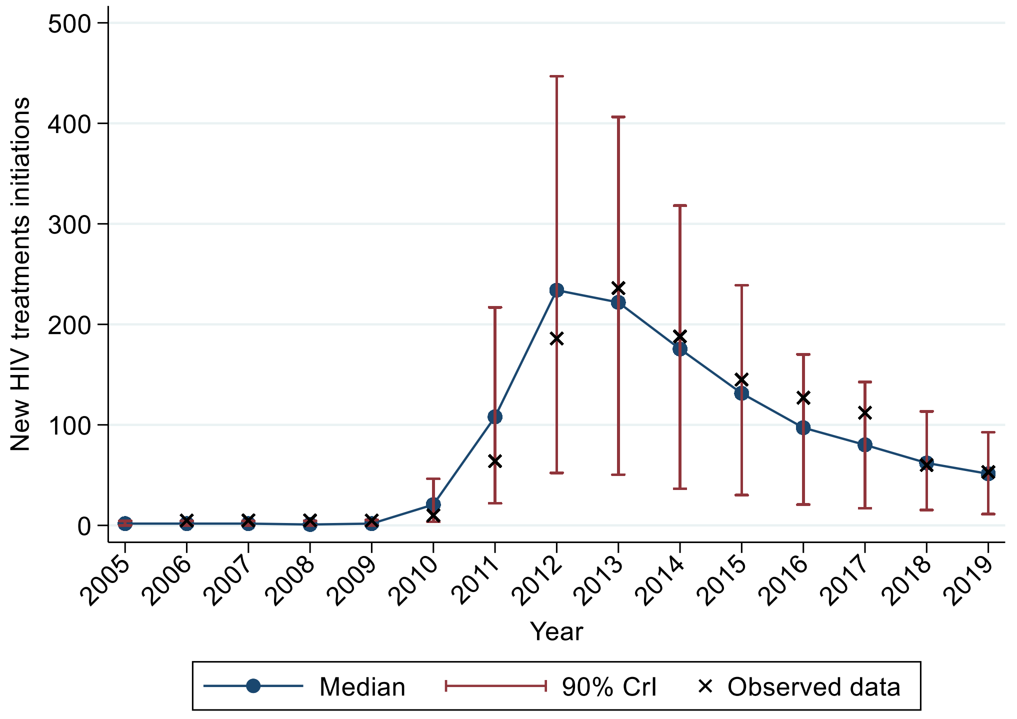

D) HIV incidence rate

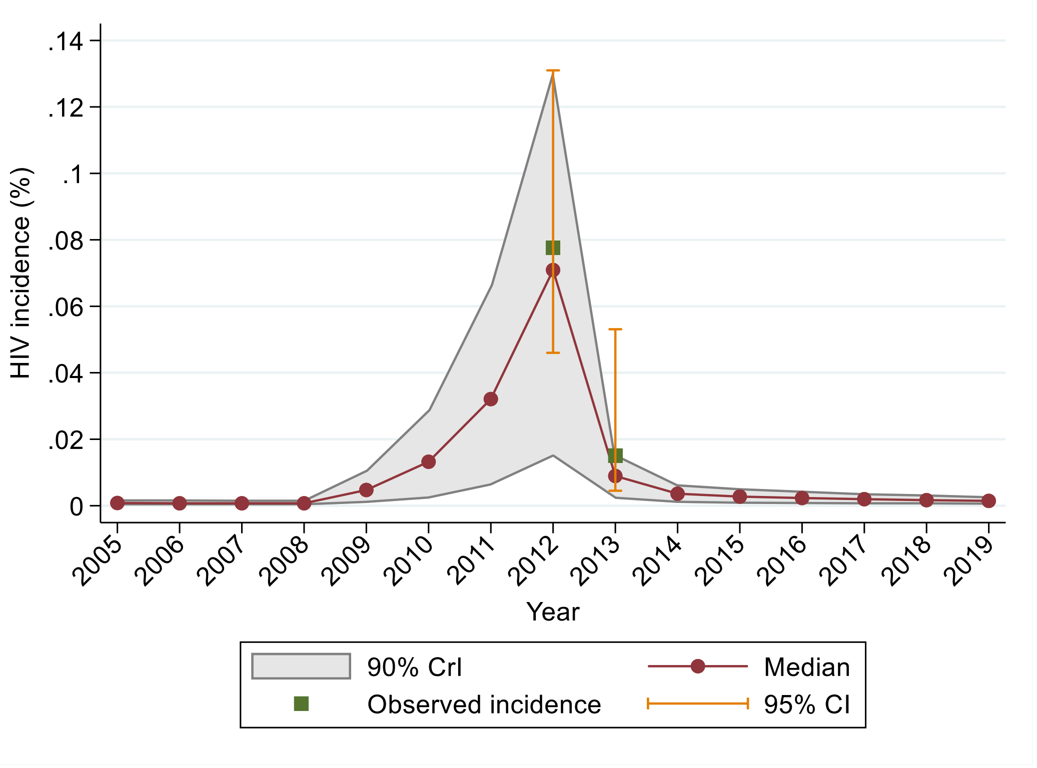
HIV incidence (%)
.14
.12
.1
.08
.06
.04
.02
0
2005
2006
2007
2008
2009
2010
2011
2012
2013
2014
2015
2016
2017
2018
2019
Year
90% CrI
Median
Observed incidence
95% CI
