# Supplementary material for: Could the 2010 HIV outbreak in Athens, Greece have been prevented? A mathematical modeling study
Source: PLoS One. 2021 Oct 7;16(10):e0258267. doi: 10.1371/journal.pone.0258267 (PMC8496824; doi:10.1371/journal.pone.0258267)

Figure S13. Estimation of the number of incident cases of HIV infection under the status quo and 1-year earlier scenario.

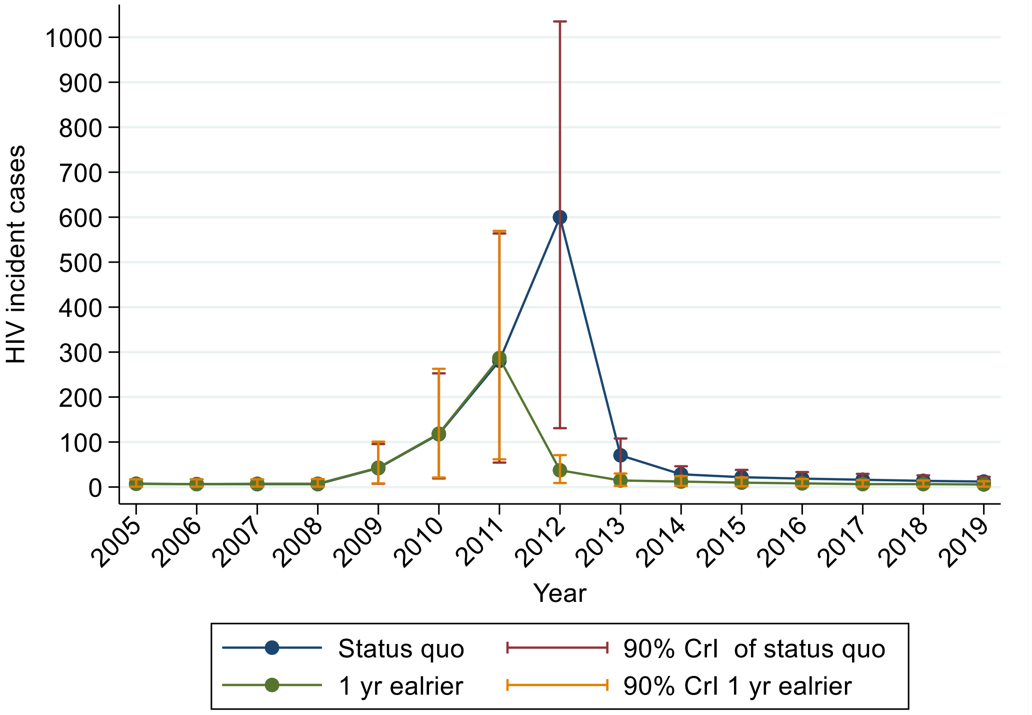

Supplement: S13 Fig — (PDF) [file pone.0258267.s014.pdf]
