# Supplementary material for: Could the 2010 HIV outbreak in Athens, Greece have been prevented? A mathematical modeling study
Source: PLoS One. 2021 Oct 7;16(10):e0258267. doi: 10.1371/journal.pone.0258267 (PMC8496824; doi:10.1371/journal.pone.0258267)
Supplement: S1 Table — (PDF) [file pone.0258267.s018.pdf]

**Table S1.** Calibration parameters

| Year | | | |
|---|---|---|---|
| | **HIV prevalence (%)** | **New diagnoses** | **New ART initiation** |
| 2006 | 0,5 | 17 | 5 |
| 2007 | 0,5 | 11 | 5 |
| 2008 | 0,5 | 11 | 5 |
| 2009 | 0,7 | 15 | 5 |
| 2010 | 0,8 | 16 | 10 |
| 2011 | 7,8 | 266 | 64 |
| 2012 | 10,8 | 551 | 186 |
| 2013 | 13,7 | 272 | 236 |
| 2014 | 10,7 | 106 | 188 |
| 2015 | 14,3 | 70 | 145 |
| 2016 | 9,2 | 81 | 127 |
| 2017 | 10,5 | 86 | 112 |
| 2018 | 7,6 | 106 | 60 |
| 2019 | | 72 | 53 |
