# Supplementary material for: Could the 2010 HIV outbreak in Athens, Greece have been prevented? A mathematical modeling study
Source: PLoS One. 2021 Oct 7;16(10):e0258267. doi: 10.1371/journal.pone.0258267 (PMC8496824; doi:10.1371/journal.pone.0258267)
Supplement: S2 Table — (PDF) [file pone.0258267.s019.pdf]

**Table S2.** Sensitivity Analysis table.

| Parameters | Value used in the primary analysis | Values examined in the sensitivity analysis |
|---|---|---|
| **Duration of injecting carrier among PWID in Athens** | 12 years | 10 or 14 years |
| **Overall PWID mortality per annum** | 2% | 1% or 4% |
| **Injection-related transmissibility while on ART compared to latent phase** | 50% | 25% or 75% |
| **Proportion of sharers PWID** | 23% | 15% or 30% |
| **PWID population size in Athens** | 9000 | 8000 vs 10000 |
| **ART discontinuation rate per year** | 6.5% | 3% or 10% |
