# Supplementary material for: Could the 2010 HIV outbreak in Athens, Greece have been prevented? A mathematical modeling study
Source: PLoS One. 2021 Oct 7;16(10):e0258267. doi: 10.1371/journal.pone.0258267 (PMC8496824; doi:10.1371/journal.pone.0258267)
Supplement: S3 Table — (PDF) [file pone.0258267.s020.pdf]

Table S3. Time to virological response during ART therapy by period.

Time	Total	Fail	Survivor Function	95% Confidence Intervals
12	130	402	0.2607	0.2243, 0.2984
24	67	55	0.1451	0.1169, 0.1784
36	27	33	0.0646	0.0477, 0.0944
